# Supplementary material for: Bacteria That Made History: Detection of Enterobacteriaceae and Carbapenemases in the Waters of Southern Brazil’s Largest Flood
Source: Microorganisms. 2025 Oct 15;13(10):2365. doi: 10.3390/microorganisms13102365 (PMC12566582; doi:10.3390/microorganisms13102365)
Supplement: Supplementary file 1 [file microorganisms-13-02365-s001.zip › microorganisms-3756731-supplementary.pdf]

Bacteria That Made History: Detection of *Enterobacteriaceae* and Carbapenemases in the Waters of Southern Brazil's Largest Flood

João Vitor Barboza Cardoso, Dariane Castro Pereira, William Latosinski Matos, Gabriela Simões de Oliveira, Victória Rodrigues de Carvalho, Louidi Lauer Albornoz, Afonso Luis Barth, Salatiel Wohlmuth da Silva, Andreza Francisco Martins.

Supplementary material

Table S1: Ct values of the samples.

	8th May 2024 to 11th May 2024			21st May 2024			6th June 2024			27th June 2024		
	Enterobacteriaceae (Ct)	*blaKPC* (Ct)	*blaNDM* (Ct)	*Enterobacteriaceae* (Ct)	*blaKPC* (Ct)	*blaNDM* (Ct)	*Enterobacteriaceae* (Ct)	*blaKPC* (Ct)	*blaNDM* (Ct)	*Enterobacteriaceae* (Ct)	*blaKPC* (Ct)	*blaNDM* (Ct)
1C (P1)	27,29	31,41	31,55	21,74	24,75	29,88	21,57	23,61	28,87	20,6	23,96	26,55
3C (P6)	22,15	28,61	31,08	31,31	30,95	und	28,69	31,36	30,21	28,69	30,3	33,91
4C (P7)	22,84	25,6	30,99	32,43	31,73	und	30,49	31,21	33,19	22,91	24,13	29,4
5C (P10)	28,19	27,97	und	29,73	30,91	32,99	29,97	29,53	31,63	33,12	31,82	und
6C (P13)	26,22	29,26	33,48	32,81	34,08	und	26,21	27,96	31,73	29,28	30,51	31,8
8C (P14)	34,02	33,65	und	31,1	28,11	30,13	29,13	29,45	30,44	29,52	29,1	30,65
8C (P16)	34,02	33,65	und	28,02	28,39	28,15	28,23	29,44	27,44			
13C (P26)				29,35	27,98	29,97	27,22	27	26,28	28,26	28,85	27,26
10C (P29)	29,22	30,46	32,12	24,29	28,09	25,21	26,65	25,71	26,7	26,72	26,97	25,32
10C (P30)	29,22	30,46	32,12	29,2	31,27	31,89	27,97	27,19	27,5	28,24	29,19	25,51
10C (P31)	29,22	30,46	32,12	29,2	27,51	30,59	29,39	28,36	29,64	28,96	28,13	30,09
9C (P32)	27,55	28,91	30,14	22,29	21,94	25,52	30,04	28,09	29,98	31,55	29,92	33,14
15C (P38)	26,33	27,3	30,4	27,65	29,13	30,06	28,77	30,6	28,08	28,35	28,63	26,59
16C (P39)	24,97	26,38	27,06	29,65	30,49	30,37	27,45	27,7	25,49	29,56	30,41	31,74
18C (P42)	28,69	30,24	und	29,58	28,39	30,63	29,28	29,85	28,91	29,91	30,21	27,99
19C (P43)	25,77	29,84	28,46	32,51	31,74	32,62	28,17	30,78	28,77	31,88	33,29	und
19C (P44)	25,77	29,84	28,46	28,47	27,42	31,69	30,39	32,06	32,03	30,84	31,95	32,96
20C (2V)	31,54	34,93	34,38				31,49	34.74	und	31,55	und	und
20C (3V)	31,54	34,93	34,38	31,22	und	und	34,02	und	und	31,6	und	32,87
20C (4V)	31,54	34,93	34,38	32,16	34,6	26,8				31,26	und	und
11C	24,08	24,65	25,05									
14C	24,59	25,81	26,51									
17C	24,05	29,36	27,95									
Itapuã D										23,37	28,45	29,37
Lami D										28,94	34,93	32,93
